# *Dinebra retroflexa* Herbal Phytotherapy: A Simulation Study Based on Bleomycin-Induced Pulmonary Fibrosis Retraction Potential in Swiss Albino Rats

**DOI:** 10.3390/medicina58121719

**Published:** 2022-11-24

**Authors:** Atef A. El-Hela, Mostafa M. Hegazy, Hatem S. Abbass, Amal H. Ahmed, Marwa S. Abu Bakr, Rawah H. Elkousy, Adel Ehab Ibrahim, Sami El Deeb, Ossama M. Sayed, Enas S. Gad

**Affiliations:** 1 Department of Pharmacognosy and Medicinal Plants, Faculty of Pharmacy, Al-Azhar University (Boys), Cairo 11884, Egypt; 2 Department of Pharmacognosy, Faculty of Pharmacy, Sinai University—Kantara Branch, Ismailia 41636, Egypt; 3 Department of Pharmacognosy and Medicinal Plants, Faculty of Pharmacy, Al-Azhar University (Girls), Cairo 11884, Egypt; 4 Natural and Medical Sciences Research Center, University of Nizwa, Birkat Al Mauz, P.O. Box 33, Nizwa 616, Oman; 5 Department of Pharmaceutical Analytical Chemistry, Faculty of Pharmacy, Port-Said University, Port-Said 42511, Egypt; 6 Institute of Medicinal and Pharmaceutical Chemistry, Technische Universitaet Braunschweig, 38092 Braunschweig, Germany; 7 Department of Pharmaceutics, Faculty of Pharmacy, Sinai University—Kantara Branch, Ismailia 41636, Egypt; 8 Department of Pharmaceutical Sciences, King Faisal University, Al-Hofuf 13890, Saudi Arabia; 9 Department of Pharmacology and Toxicology, Faculty of Pharmacy, Sinai University—Kantara Branch, Ismailia 41636, Egypt

**Keywords:** *Dinebra retroflexa*, bleomycin, pulmonary fibrosis, silver nanoparticles, UHPLC/QTOF-MS/MS

## Abstract

*Background and Objectives:* Fibrotic lung disease is one of the main complications of many medical conditions. Therefore, the use of anti-fibrotic agents may provide a chance to prevent, or at least modify, such complication. The aim of this study was to evaluate the protective pulmonary anti-fibrotic and anti-inflammatory effects of *Dinebra retroflexa. Materials and methods: Dinebra retroflexa* methanolic extract and its synthesized silver nanoparticles were tested on bleomycin-induced pulmonary fibrosis. Pulmonary fibrosis was induced by intratracheal instillation of bleomycin (5 mg/5 mL/kg-Saline) as a supposed model for induced lung fibrosis. The weed evaluation was performed by intratracheal instillation of *Dinebra retroflexa* methanolic extract and its silver nanoparticles (35 mg/100 mL/kg-DMSO, single dose). *Results:* The results showed that both *Dinebra retroflexa* methanolic extract and its silver nanoparticles had a significant pulmonary fibrosis retraction potential, with Ashcroft scores of three and one, respectively, and degrees of collagen deposition reduction of 33.8 and 46.1%, respectively. High-resolution UHPLC/Q-TOF-MS/MS metabolic profiling and colorimetrically polyphenolic quantification were performed for further confirmation and explanation of the represented effects. Such activity was believed to be due to the tentative identification of twenty-seven flavonoids and one phenolic acid along with a phenolic content of 57.8 mg/gm (gallic acid equivalent) and flavonoid content of 22.5 mg/gm (quercetin equivalent). *Conclusion: Dinebra retroflexa* may be considered as a promising anti-fibrotic agent for people at high risk of complicated lung fibrosis. The results proved that further clinical trials would be recommended to confirm the proposed findings.

## 1. Introduction

Pulmonary fibrosis is one of the clinical conditions that represent a major cause of mortality [1]. For instance, during the COVID-19 pandemic, it was discovered that the human lung was considered the most affected organ by SARS-CoV-2 virion [2]. Anti-fibrotic drugs could have therapeutic potential for treating severe pulmonary fibrosis complications and preventing the long-term fibrotic consequences [3]. Although several chemical molecules have been developed for treatment of pulmonary fibrosis, some drugs could have potent side effects such as reducing the function of lungs, or patients could have different clinical complications owing to their heterogeneity [4]. Therefore, the combination of drugs, especially with conventional herbal treatments, is a novel measure for avoiding the complications of this deadly disease. It is well known that the use of herbal treatment can lead to drug dose re-adjustment which can provide acceptable therapeutic effect with minimal noisy side-effects [4]. Several articles have studied the effect of different plants as anti-fibrotic drugs [5,6]. Interestingly, there are not enough studies or research on the expectable pharmacological effect of *Dinebra retroflexa* (DR) on different pathological conditions, although the family Poaceae is one of the most ecologically, economically, and medicinally important plant families. Poaceae plants were reported as a folk medicine for different disease prevention and/or treatment alongside isolation of different phytoconstituents [7]. DR is a cattle fodder invasive annual weed with densely tufted grass native to tropical Asia, the Middle East, and parts of Africa, and known as Deneib or Negeil El-Nimr. DR on the one hand can cause serious problems and on the other hand is used for various purposes by local people [8]. DR is commonly recorded in fields of soybean, cotton, and maize causing yield reduction via competing for light, moisture, nutrients, and space. DR is considered to be an excellent fodder for cattle, used for grazing and for increasing the milk flow but not suitable for making hay for ensilage, which is encouraging for its possible safety for humans [9,10,11]. DR requires management and control, but the continuous overuse of herbicides has led to rapid evolution of herbicide resistance and exacerbated weed problems [12,13].

One of the most famous experimental models for studying lung fibrosis is the bleomycin (BLM) induced model in rats [14]. Although interstitial lung fibrosis is a common side effect of BLM, it is still considered a well-established and commonly used model for induction of pulmonary fibrosis in rodents. This could be attributed to the fact that some of the molecular signatures and some histopathological hallmarks resemble those encountered in human fibrotic lung diseases [15]. Meanwhile, silver nanoparticles (Ag-NPs) are a versatile class of engineered nanomaterials, used in a wide range of applications (biomedical applications, pharmaceutical products, cosmetics, textiles, paints, coatings, clean water technology, climate change and contamination control, and energy generation) [16]. Plant-mediated biological synthesis of nanoparticles is gaining importance due to its simplicity, eco-friendliness, cost-effectiveness, and easy scalability for vast scale synthesis [16]. The plasma membrane’s permeability for small-sized Ag-NPs pharmaceutical products allows their accumulation inside the cell through phagocytosis, endocytosis, or micropinocytosis uptake mechanisms in a dose-dependent manner. The smaller the Ag-NPs, the more they can interact with cell surfaces and penetrate and pass through the organism’s membranes [17].

The proposed study is aimed at exploring the capabilities of DR in improving lung function and retracting progressive fibrosis, as DR might represent a promising support within the treatment protocols for improving the lung function in several associated conditions, such as in COVID-19 infections. This paper also describes the protective anti-fibrotic and anti-inflammatory effects of *Dinebra retroflexa* methanolic extract (DRE) and *Dinebra retroflexa* methanolic extract loaded on Ag-NPs (DRN) on retraction of lung fibrosis in BLM-injected rats as a supposed model of pulmonary fibrosis. In addition, this paper describes the quantitative determination of polyphenolics colorimetrically, and illustrates the bioactive secondary metabolites responsible for detected activities using ultra-high performance liquid chromatography/quadrupole time-of-flight mass spectrometer analysis (UHPLC/QTOF-MS/MS) as a reliable, fast, accurate, simple, and reproducible technique.

## 2. Materials and Methods

### 2.1. Extraction of Plant Material

The aerial parts (linear leaves, inflorescence, and flowers) of *Dinebra retroflexa* (Vahl) Panz. (family Poaceae) were collected from the Tanta region (Ekhnawy) in Egypt in June 2020. The plant material was authenticated in the Botany Department, Faculty of Science, Al-Azhar University, Cairo, Egypt. A specimen of the aerial parts of DR was deposited in the Pharmacognosy Department, Faculty of Pharmacy, Al-Azhar University, Cairo, Egypt. The aerial parts were air dried under shade at room temperature and powdered to afford 480 g. Soxhlet apparatus using 1 L of 70% methanol was used for the extraction process to afford 63 g. The methanolic extract was then evaporated under vacuum by rotatory evaporator (BUCHI Rotavapor^®^ model R-210/R-215, Darmstadt, Germany). The dry residues were then used for the green synthesis of Ag-NPs, quantitative determination of polyphenolics, UHPLC/QTOF-MS/MS analysis, and in vivo investigations of BLM-induced lung fibrosis retraction.

### 2.2. Synthesis of Ag-NPs

For the synthesis of Ag-NPs, silver nitrate solution (5.0 mM) (Sigma-Aldrich, GMbH, Germany) was used. An 8% (*w*/*w*) sodium citrate solution (2.0 mM) (Sigma-Aldrich, GMbH, Germany) was used as a reducing agent for synthesis of Ag-NPs while an aqueous solution of DRE prepared at a concentration of 0.1 g/mL was used as a reducing and capping solution for synthesis of DRN at room temperature. Briefly, each solution was added to separate flasks containing silver nitrate in stirring condition (700 rpm). The stirring continued until the transparent colorless solutions were converted to a characteristic pale yellow and pale red color indicating the formation of Ag-NPs and DRN, respectively. The nanoparticles were purified by centrifugation and the precipitates were washed at least three times with deionized water under nitrogen stream to remove excess silver ions. Concerning TEM size and imaging characterization, the Ag-NPs powders were obtained by freeze-drying and then re-suspended in deionized water and homogenized with an ultrasonic cleaning container (Fisher Bioblock Scientific, Surry, UK). Images were obtained using JEOL JEM-1010 TEM (JEOL Ltd., Tokyo, Japan) at 80 KV, at the Regional Center for Mycology and Biotechnology (RCMB), Al-Azhar University.

### 2.3. Lung Fibrosis Modality

#### 2.3.1. Animals

Swiss albino adult male rats weighing 180–200 g were obtained from the Theodor Bilharz Research Institute (Giza, Egypt). Animals were kept in standard conditions of temperature (22 °C ± 0.5 °C) and relative humidity (55 ± 1), with a 12 h light/12 h dark cycle at the animal house facility of the Faculty of Pharmacy, Suez Canal University (Ismailia, Egypt). Rats were fed a standard chow diet composed of 20% casein, 15% corn oil, 55% corn starch, 5% salt mixture, and 5% vitaminized starch (Egyptian Company of Oils and Soap, Kafr-Elzayat, Egypt) and had free access to water *ad libitum* throughout the experiment. All animals’ procedures were performed in accordance with the Ethics Committees of the Faculty of Pharmacy, Suez Canal University, Ismailia, Egypt. This research approval was encoded 202112RA1; Dated December 2021.

#### 2.3.2. Induction of Pulmonary Fibrosis

To induce pulmonary fibrosis, bleomycin was injected in rats through intratracheal instillation from BLEOCIP 15^®^ vials (Cipla Pharmaceuticals, Mumbai, India). The rats were first anesthetized using Thiopental sodium^®^ vials (supplied by EIPI Co., Tenth of Ramadan City, Sharkia, Egypt) at a dose of 50 mg/kg; I.P. [18]. Then bleomycin was injected at a dose of 5 mg/kg with an adjusted volume of 5 mL/kg [19]. During instillation, rats were allocated in vertical positions and their bodies were finely flipped to the right and left sides to ensure the complete and uniform distribution of BLM within the lung tissues.

#### 2.3.3. Experimental Design

A total of 60 male Swiss albino rats were allotted. All rats were weighed and randomly allocated into six groups.

- Control group: 10 Rats were injected with the drug vehicle (saline) in a dosing volume of (5 mL/kg, IT).
- Drug control group: 10 Rats were injected with a single dose of DRE (35 mg/100 mL/kg-DMSO, IT).
- BLM group: 10 Rats were injected with a single dose of BLM (5 mg/5 mL/kg-Saline, IT).
- DRE group: 10 Rats were injected with a single dose of DRE (35 mg/100 mL/kg-DMSO, IT) after one hour of BLM instillation.
- DRN group: 10 Rats were injected with a single dose of DRN (35 mg/100 mL/kg-DMSO, IT) after one hour of BLM instillation.
- Ag-NPs group: 10 Rats were injected with a single dose of Ag-NPs prepared by citrate reduction (35 mg/100 mL/kg-DMSO, IT) after one hour of BLM instillation.

At the end of the experiment (14 days), retro-orbital blood samples were withdrawn from the orbital plexus under anesthesia (thiopental sodium 50 mg/kg, IP) [18] using heparinized micro-capillaries (Optilab, Berlin, Germany). Serum was separated by blood centrifugation at 4000 rpm for 10 min at -4^◦^C (Heraeus Biofuge, Berlin, Germany). After terminal bleeding, animals were euthanized by cervical dislocation, and the lungs were dissected out, washed with normal saline and blotted dry on filter paper. The lungs were preserved in 10% formol saline and used thereafter for histological and immune–histochemical examinations.

#### 2.3.4. Lung Histopathology

Lungs were kept in 10% formol-saline for 24 h using Hartz Technique (1947) [20]. Tissues were then embedded in paraffin blocks, and 5 micron-thick sections were obtained from the blocks and stained by H&E and Masson Trichrome. The tissue sections were then examined microscopically by a pathologist. The tissues were examined under a microscope in a random order and the pathologist was blinded against the tissues of the experimental groups in order to minimize bias [21]. The structural alterations of tissue were assessed based on the alveolar wall thickening, presence of fibrotic foci, inflammatory lesions, and collagen deposition [22]. The leveling of lung fibrosis in the lung sections stained with H&E was determined quantitatively using the Ashcroft scoring evaluation, however, fibrosis in the Masson Trichrome stained lungs was quantitatively analyzed by measuring the intensity of collagen deposition in the lungs using the image J software which was developed by the National Institute of Health (NIH Image J, Version 1.31, Bethesda, MD, USA). Data were expressed as OD.

#### 2.3.5. Determination of the Intensity of Lung Fibrosis Using the Modified Ashcroft Score Technique

Fibrosis was quantified using a modified Ashcroft scale designed for a standardized fibrosis evaluation in small animals [23]. Images were analyzed by two investigators unaware of the type of treatment, and the severity of fibrosis was assessed according to the method proposed by Ashcroft et.al. [24]. Briefly, the grade of lung fibrosis was scored on a scale from 0 to 8 by examining 6 randomly selected fields per section and then calculated.

The criteria for grading lung fibrosis were as follows:

- Grade 0: Normal lung tissue.
- Grade 1: Minimal fibrous thickening of alveolar or bronchiolar walls.
- Grade 2: Mild fibrous thickening of alveolar or bronchiolar walls.
- Grade 3: Moderate thickening of walls without obvious damage to lung architecture.
- Grade 4: Increased fibrosis with focal damage to lung structure and formation of fibrous bands or single fibrous nodule.
- Grade 5: Increased fibrosis with definite damage to lung structure and formation of fibrous bands or small fibrous nodules.
- Grade 6: Wide fibrosis with wide damage to lung structure and formation of fibrous bands or fibrous masses.
- Grade 7: Severe distortion of structure and large fibrous areas.
- Grade 8: Total fibrous obliteration of the fields.

#### 2.3.6. Statistical Analysis

All results were tabulated and expressed as mean ± S.E.M. Results were assessed by one-way repeated measures analysis of variance (ANOVA) followed by Tukey’s post hoc test. Data were analyzed using the statistical package for the social sciences, version 20 (SPSS Inc., Chicago, IL, USA). A *p* value < 0.05 was considered to be statistically significant, and all possible comparisons were made among the study groups.

### 2.4. UHPLC/Q-TOF-MS-MS Metabolic Profiling

Separation of phytoconstituents was performed on an Axion AC system (Kyoto, Japan) connected with an auto sampler system, an in-line filter disks pre column (0.5 µm × 3.0 mm, Phenomenex, Torrance, CA, USA), and a Xbridge C18 (3.5 µm, 2.1 × 50 mm) column (Waters Corporation, Milford, MA, USA) [25]. Mass spectrometry was performed on a Triple TOFTM 5600 system quadrupole-time-of-flight mass spectrometer (QTOF/MS) with a Duo-Spray TM source operating in the ESI mode (AB SCIEX, Concord, Toronto, ON, Canada), with sprayer capillary and de-clustering potential voltages of 4500 and 80 V, respectively. The method consisted of high-resolution survey spectra at which mobile phase composition, gradient elution, source temperature, collision energy, ion tolerance, operation parameters, and the database search parameters’ settings were performed according to the method discussed in our previous publication [25].

### 2.5. Quantitative Determination of Polyphenolics

Quantitative determination of phenolic and flavonoid contents of DRE were estimated colorimetrically according to a previously published method [26] using a Genesys Spectrophotometer (Milton Roy, INC., Rochester, NY, USA). The reagents used for each were Folin–Ciocalteu (Sigma Chemical Co., St. Louis, MO, USA) and Gallic acid (Merck, Darmstadt, Germany) for phenolic content quantification, and Quercetin (Merck Co., Darmstadt, Germany) and Aluminum chloride (Merck, Darmstadt, Germany) for flavonoid content quantification.

## 3. Results

### 3.1. Synthesis of Ag-NPs

**Figure 1:**
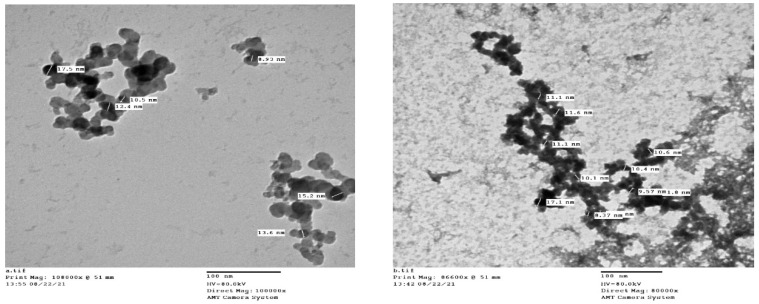
TEM images of Ag-NPs prepared by citrate reduction (**a**) and DRE reduction (**b**).

Transmission electron microscope (TEM) images showed Ag-NPs prepared by citrate reduction and DRN prepared by DRE reduction and stabilization (Figure 1). Ag-NPs and DRN appeared as well-defined spherical particles in aggregates. The DRN size measurement achieved smaller particle size (8.95 ± 3.3 nm) compared to citrate-produced Ag-NPs (13.1 ± 2.6 nm). This finding was in accordance with previous literature that reported the efficiency of plant extract to produce NPs with smaller particle sizes than chemical procedures [16,27,28].

### 3.2. Effect of DRE and DRN on Lung Histopathology and Aschroft Fibrosis Score in BLM Male Swiss Albino Rats

**Figure 2:**
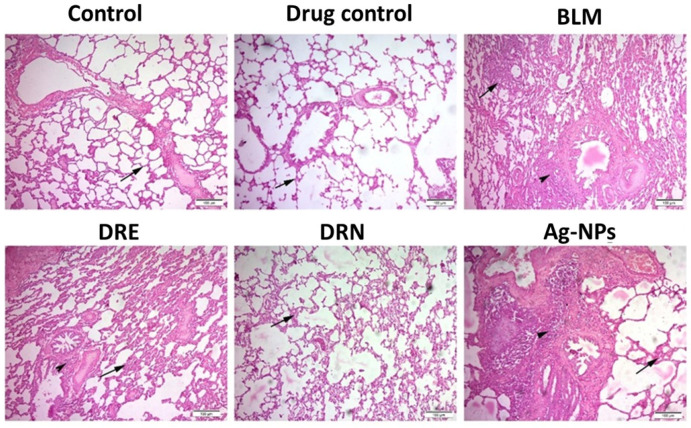
Effect of DRE and DRN on lung histopathology in BLM male Swiss albino rats (H&E × 100; image resolution 300 DPI).

Hematoxylin and Eosin staining (H&E) observations on lung sections from both the normal control and drug control groups had normal histological structures of the inter-alveolar septa and lung alveoli. However, lung sections from the BLM group showed congestion of the peri-alveolar and peri-bronchial blood capillaries, areas of emphysema with distributed inflammatory cells with proliferation of the alveolar epithelium, and had the highest mean Ashcroft fibrosis score (6 ± 0.22). Lungs of the DRE group showed obvious retraction of inflammation with mild to moderate alveolar thickening and the mean Ashcroft score was decreased to grade 3 compared to the BLM treated rats. However, the intratracheal instillation (IT) of DRN showed great therapeutic effect with large retraction of fibrosis induced by BLM and very few inflammatory cells, with clear thinning of the alveolar wall in the DRN group. The mean Ashcroft score in this group was decreased to grade 1 compared to the BLM group. Lungs of the Ag-NPs group and its mean Ashcroft scoring resembled that of the BLM group (Figure 2 and Table 1).

The lungs of the control group and drug control group showed normal histological morphology of the alveoli and inter-alveolar septa. The lungs of the BLM group showed great congestion of the alveolar wall (arrow) with distributed inflammatory cells in the inter-alveolar septa (arrowhead). The lung tissues of the DRE group showed a moderate retraction of the alveolar thickness (arrow), and around the alveoli (arrowhead). However, those of the DRN group showed a marked retraction of fibrosis with very few inflammatory cells (arrow). The lung sections of the Ag-NPs group presented the same as the lung sections of the BLM challenged rats.

### 3.3. Effect of DRE and DRN on Collagen Disposition in the Lungs of BLM Male Swiss Albino Rats

**Figure 3:**
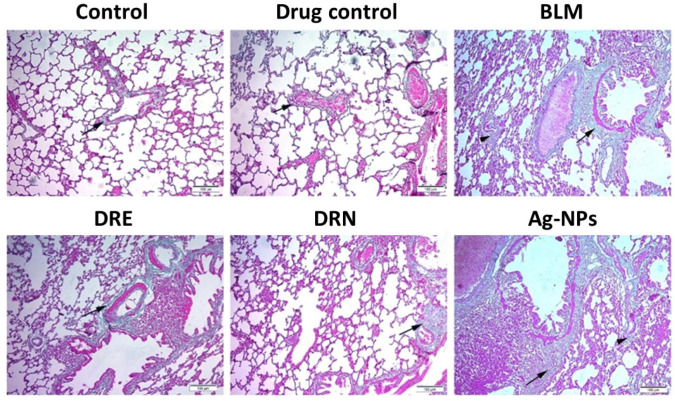
Effect of DRE and DRN on collagen deposition in the lungs of BLM male Swiss albino rats (Masson Trichrome × 100; image resolution 300 DPI).

Masson Trichrome staining observations on lung sections from both the control and drug control groups showed fine collagen fiber deposition in the intra-alveolar walls and around alveoli with normal optical densities (ODs). The lung sections of the BLM group showed excessive collagen deposition around the alveoli, with marked thickening of the alveolar wall and with ODs increased by 96.5% compared to the control group. The lung tissues of the DRE group showed moderately high reduction in the collagen deposition in the intra-alveolar septa and a significant decrease of 33.8% in the ODs compared to the BLM group. The lung sections treated with DRN showed marked retraction of collagen deposition around the alveoli, with marked thinning of the alveolar wall and with a significant reduction of 46.1% in the ODs compared to the BLM group. The lungs of the Ag-NPs group showed nearly the same results as the BLM group (Figure 3 and Table 1).

The lungs of the control group and drug control group showed fine deposition of collagen fibers in the intra-alveolar septa with normal thickness to the alveolar wall (arrow). The lungs of the BLM group showed marked deposition of the collagen around the alveoli (arrow) and in the intra-alveolar septa (arrowhead). The lung tissue of the DRE group showed a moderate thickness to the alveolar wall with moderate collagen deposition (arrow); however, those of the DRN group showed a retraction of the collagen deposition in the intra-alveolar septa (arrow). The lung sections of the Ag-NPs group showed the same results as the lung sections of BLM challenged rats.

### 3.4. UHPLC/Q-TOF-MS-MS Metabolic Profiling

Data were processed by MS-DIAL 3.96 software for non-targeting small molecule comprehensive analysis of the sample. The phytochemical constituents of DRE were analyzed via UHPLC/Q-TOF-MS-MS in positive ionization mode. A total of twenty-eight identified peaks were listed according to their retention times (Table 2). The tentative identification revealed the presence of one phenolic acid (Chlorogenic acid (1)) and twenty-seven flavonoid compounds including twelve flavonols (Rutin (6), Spiraeoside (10), Quercetin-3-O-Glucuronide (11), Astragalin (12), Isorhamnetin-3-O-glucoside (13), Afzelin (14), Narcissin (16), Kaempferol (17), Quercetin (18), Quercitrin (19), Vincetoxicoside B (21), and Kaempferide (24)); ten flavones (Luteolin 8 C-hexosyl- O-hexoside (2), Saponarin (3), Apigenin 6-C-glucoside 8-C-xyloside (4), Apigenin O-pentosyl-C-hexoside (5), Apigenin 6-C glucoside (7), Cosmosiin (9), Luteolin-7-O-glucoside (15), Neodiosmin (20), Tricin (23), and Acacetin (26)); four isoflavones (Sophoricoside (8), Formononetin (25), Calycosin (27), and Biochanin A (28)); and one anthocyanin (Malvidin-3-O-glucoside (22)). The compounds’ identification was based on the data obtained from PCA scores’ plot analysis from MS-DIAL [29]. To improve mass accuracy, only the compounds with highest total score above 70% were assigned. Furthermore, the high-resolution mass error values were calculated according to the formula: [Error = (Experimental mass − Calculated mass)/Experimental mass] × 10^6^ at which compounds with high-resolution mass error of more than 14 ppm were excluded [25]. Further confirmation of the compounds was carried out by comparing their data with the molecular ion masses and characteristic mass fragments from the ReSpect databases and the literature mass values are represented along with their literature references (Table 2). Flavonoids were the most abundant compounds, which include either aglycones (8 compounds) or glycosides (O-glycosides, C-glycosides, and hybrids of both, 19 compounds) as protonated molecules [M+H]^+^. Elimination of 176, 162, 146, and 132 atomic mass unit (amu) fragments corresponded to flavonoid O-glycosides, upon the loss of hexuronic acid, hexose, deoxy hexose, and pentose moieties, respectively. In contrast, characteristic neutral losses of 150, 120, and 90 amu for C-hexosides; 90 and 60 amu for pentose residues; and 18, 36, and 54 amu for one, two, and three water molecule(s) were characteristic for flavonoid C-glycosides [30]. Moreover, flavonoid aglycones underwent further fragmentation by losing 15, 18, 28, and 42 amu for (CH_3_), (H_2_O), (CO), and (CH_2_CO), respectively. Fragmentation of flavonoid C-ring by the retro Diels –Alder (RDA) cleavage mechanism gave an idea about the hydroxylation patterns since fragments of 153 and 137 amu, and 137 and 121 amu for A-ring and B-ring with two and one hydroxy substitutions, respectively, were represented for each compound (Table 2) [31].

### 3.5. Quantitative Determination of Polyphenolics

The total phenolic content value was 57.8 ± 0.42 mg/gm dry extract and expressed as gallic acid equivalent (GAE). While the total flavonoid content was 22.5 ± 0.32 mg/gm dry extract and expressed as quercetin equivalent (QE).

## 4. Discussion

Pulmonary fibrosis is a devastating lung disease characterized by a progressive decline in lung function which consequently can result in death, as demonstrated by several conditions, such as COVID-19 infection [3]. Anti-fibrotic drugs could have therapeutic potential for treating severe pulmonary fibrosis complications and preventing long-term fibrotic consequences [3]. Herbal medicine is a type of traditional medicine that is approved for treatment of several diseases, or at least to improve health and prevent illness. Several articles have focused on the anti-fibrotic effects of different plants [5,6]. Weeds are plants that appear without being sown or cultivated in areas that are otherwise managed or controlled. The medicinal use of weeds is gaining importance as they are prevalent and inexpensive resources, and it could provide an economic and eco-friendly weeds management strategy [53].

The current work studied the effect of DR plant on improving the lung fibrosis induced by BLM in male Swiss albino rats. BLM is a chemotherapeutic antibiotic that was originally extracted from *Streptomyces verticllus* [54]. It has been used for the treatment of cancers of the skin, head and neck, oesophagus, and penis and testes, as well as for Hodgkin’s and non-Hodgkin’s lymphoma [55]. Interstitial lung fibrosis is a common side effect of BLM, developed as a result of repeated cascades of vigorous inflammatory reactions leading to lung scarring and stiffness (such as in COVID-19) [3,56]. Cytokines such as interleukin-6 (IL-6), tumor necrosis factor-alpha (TNF-α), macrophages, platelet-derived growth factor (PDGF), and transforming growth factor-beta (TGF-β) are released from alveolar macrophages in the animal models of BLM toxicity, resulting in fibrosis [57]. A vigorous interaction between the released inflammatory cytokines and the alveolar epithelial cells leads to further release of TGF-β and other inflammatory cytokines and, again, to a cycle of repeated pathological series leading to lung scarring, stiffness, and fibrosis [55]. All of these parameters are released during the inflammatory state and during fibrosis development of the tissue. Although TNF-α and IL-6 are anti-inflammatory cytokines, they are not produced in enough concentration relative to the other toxic parameters. The remaining parameters are toxic to the cell and are involved in the fibrosis development pathways. Such a mechanism resembles that of lungs infected with COVID-19 and BLM induced lung fibrosis could be used as a supposed model for COVID-19 induced lung fibrosis [3,56].

The H&E histopathological observations and Ashcroft scoring of the lung sections of BLM rats treated with either DRE or DRN revealed anti-inflammatory and anti-fibrotic effects. Both DRE and DRN showed significant pulmonary fibrosis retraction potential, however, DRN showed greater retraction potential compared to DRE. Regarding Masson Trichrome staining observations, DRE was effective in ameliorating the excessive collagen deposition in the intra-alveolar septa, however, DRN showed greater retraction potential compared to DRE. This finding agreed with previous literature which stated that the predominant accumulation of a plant extract loaded on Ag-NPs in the target tissue has more potent therapeutic effect than the use of plant extract alone [58]. The plasma membrane’s permeability for small-sized Ag-NPs pharmaceutical products allow their accumulation inside cells through phagocytosis, endocytosis, or micropinocytosis uptake mechanisms in a dose-dependent manner. The smaller the Ag-NPs, the more they can interact with cell surfaces and penetrate and pass through the organism’s membranes [17].

Finally, DRN showed a great therapeutic potential in retraction of the BLM induced pulmonary fibrosis, which suggests that DR may have an aiding role in improving the symptoms of pneumonia observed in many patients. The anti-fibrotic and anti-inflammatory properties of DR are believed to be mainly due to its relatively high flavonoid and phenolic contents [14,59,60,61]. Flavonoids attenuate lung fibrosis by enhancing the activities of antioxidant enzymes and modulating inflammation and allergic reactions, as reported for Quercetin, Isorhamnetin, Kaempferol [60,62,63], Apigenin [64], Rutin [65], Luteolin [66], and Sophoricoside [67], which were detected along with their conjugates in DRE. The DRE detected peaks 3, 4, 5, 7, 9, and 26 were assigned for Apigenin and its conjugates; 6, 10, 11, 18, 19, and 21 were assigned for Quercetin and its conjugates; 12, 14, 17, and 24 were assigned for Kaempferol and its conjugates; 13 and 16 were assigned for Isorhamnetin conjugates; 2 and 15 were assigned for Luteolin conjugates, and 8 was assigned for Sophoricoside. In addition, specific amelioration mechanisms, including suppressing the myofibroblasts, decreasing lysosomal proteases, endoplasmic reticulum stress inhibition, attenuation of monocrotaline-induced pulmonary arterial hypertension, remitting the airway smooth muscle cell proliferation in chronic asthma, and epithelial–mesenchymal transition repression, were reported for the detected peaks: (1) chlorogenic acid [68], (20) Neodiosmin [69], (22) Malvidin-3-O-glucoside [70], (23) Tricin [71], (25) Formononetin [72], (27) Calycosin [73], and (28) Biochanin-A [74]. The role of the total polyphenols and flavonoids is collective, which might be different from and more synergistic than that of individual components.

## 5. Conclusions

In the present research, *Dinebra retroflexa* extract was investigated and evaluated for its anti-fibrotic and anti-inflammatory effect on bleomycin-induced pulmonary fibrosis in rats. The metabolic qualitative profiling of the extract under study revealed twenty-seven flavonoidal compounds and one anthocyanin compound. The histopathological findings, Ashcroft scoring evaluation, and the degree of collagen deposition expressed as OD revealed that *Dinebra retroflexa* had a potent protective effect through preventing the progression of lung damage in the model under study. The extract showed significant pulmonary fibrosis retraction potential. Such findings suggest that *Dinebra retroflexa* extract and its silver nanoparticles could have a significant therapeutic effect on the inflammation and fibrosis observed in patients’ lungs. Consequently, this study might be considered as a start, and further experimental and subsequent clinical studies will be required to confirm or deny the suggested hypothesis.

## Figures and Tables

**Table 1:** Effect of DRE and DRN on lung histopathology scoring and collagen deposition in BLM male Swiss albino rats.

Groups	Parameter	
	H&E (Ashcroft Score)	Masson Trichrome (OD)
Control group	0 ± 0.18	82.29 ± 2.75
Drug control group	0 ± 0.00	93.81± 3.32
BLM group	6 ± 0.22 ^ab^	161.66 ± 2.39 ^ab^
DRE group	3 ± 0.18 ^abc^	106.95 ± 5.63 ^abc^
DRN group	1 ± 0.22 ^cd^	87.15 ± 3.44 ^cd^
Ag-NPs group	6 ± 0.22 ^abde^	158.26 ± 3.09 ^abde^

Data are expressed as mean ± standard error, N = 6 random fields. Multiple comparisons were accomplished using one-way ANOVA test followed by Tukey’s post-hoc test. ^a^ Significantly different from the control group (*p <* 0.05). ^b^ Significantly different from the drug control group (*p <* 0.05). ^c^ Significantly different from the BLM group (*p <* 0.05). ^d^ Significantly different from the DRE group (*p <* 0.05). ^e^ Significantly different from the DRN group (*p <* 0.05).

**Table 2:** DRE bioactive constituents identified using UHPLC/QTOF-MS/MS analysis in positive ionization mode.

	*R_t_* (min)	Precursor/Adduct Ion	Error (ppm)	Characteristic Fragmentation	Proposed Compound	Identification References
1	1.23	355.1029 [M+H]^+^	0	355[M+H]^+^ 193[M+H-caffeoyl moiety]^+^ 163[M+H-Quinic acid]^+^	Chlorogenic acid	[32]
2	1.65	611.1608 [M+H]^+^	−0.7	611[M+H]^+^ 431[M+H-glucose-H_2_O]^+^ 413[M+H-glucose-2H_2_O]^+^ 395[M+H-glucose-3H_2_O]^+^	Luteolin 8 C-hexosyl-O-hexoside	[33]
3	1.94	595.1660 [M+H]^+^	−0.5	595[M+H]^+^ 577[M+H-H_2_O]^+^ 433[M+H-glucose]^+^ 415[M+H-glucose- H_2_O]^+^ 379[M+H-glucose-3H_2_O]^+^ 271[M+H-2glucose]^+^	Isovitexin-7-O-glucoside (Saponarin)	[34]
4	2.85	565.1555 [M+H]^+^	−3.5	565[M+H]^+^ 547[M+H-H_2_O]^+^ 529[M+H-2H_2_O]^+^ 511[M+H-3H_2_O]^+^ 469[M+H-60-2H_2_O]^+^ 457[M+H-90-H_2_O]^+^ 445[M+H-120]^+^ 427[M+H-120-H_2_O]^+^ 409[M+H-120-2H_2_O]^+^	Apigenin 6-C-glucoside 8-C-xyloside	[35]
5	3.27	565.1562 [M+H]^+^	0.9	565[M+H]^+^ 313[M+H-pentose-120]^+^ 283 [M+H-pentose-150]^+^	Apigenin O-pentosyl-C-hexoside	[30]
6	3.49	611.1603 [M+H]^+^	−1.5	611[M+H]^+^ 303 [M+H-rutinoe]^+^ 465 [M+H-rhamnose]^+^ 285[M+H-rhamnose-H_2_O]^+^ 153 ^1,3^A^+^	Rutin	[31,36]
7	3.51	433.1131 [M+H]^+^	−0.9	433[M+H]^+^ 415[M+H-H_2_O]^+^ 397[M+H-2H_2_O]^+^ 313[M+H-120]^+^ 283[M+H-150]^+^	Apigenin 6-C glucoside	[37]
8	3.53	433.1134 [M+H]^+^	−0.2	433[M+H]^+^ 271[M+H-glucose]^+^ 197 [M+H-glucose-2CO-H_2_O]^+^ 159 [M+H-glucose-2CH_2_CO-CO]^+^	Genistein-4’-O-glucoside(Sophoricoside)	[38]
9	3.54	433.1135 [M+H]^+^	0	271[M+H-glucose]^+^ 433[M+H]^+^ 153 ^1,3^A^+^	Apigenin-7-O-glucoside (Cosmosiin)	[39]
10	3.63	465.1031 [M+H]^+^	−0.4	465[M+H]^+^ 303[M+H-glucose]^+^ 229[M+H-glucose-H_2_O-2CO]^+^ 153 ^1,3^A^+^ 137 ^0,2^B^+^	Quercetin 4’-O-β-D-glucopyranoside (Spiraeoside)	[31,40]
11	3.71	479.0828 [M+H]^+^	0.4	479[M+H]^+^ 303[M+H-glucuronic acid]^+^ 229 [M+H-glucuronic acid-H_2_O-2CO]^+^ 153 ^1,3^A^+^ 137 ^0,2^B^+^	Quercetin-3-O-Glucuronide	[31,41]
12	3.84	449.1080 [M+H]^+^	−0.9	449[M+H]^+^ 287[M+H-glucose]^+^	Kaempferol 3-O-glucoside (Astragalin)	[42]
13	3.88	479.1193 [M+H]^+^	0.6	302[M+H-glucose-CH_3_]^+^ 317[M+H-glucose]^+^ 479[M+H]^+^	Isorhamnetin-3-O-glucoside	[43]
14	3.92	433.1154 [M+H]^+^	4.4	433[M+H]^+^ 287[M+H-rhamnose]^+^	Kaempferol-3-O-α-L-rhamnoside (afzelin)	[36]
15	4.02	449.1083 [M+H]^+^	−0.2	449[M+H]^+^ 287[M+H-glucose]^+^	Luteolin-7-O-glucoside	[44]
16	4.23	625.1757 [M+H]^+^	−1.9	317[M+H-Rutinose]^+^ 479[M+H-Rhamnose]^+^ 625[M+H]^+^	Isorhamnetin-3-O-rutinoside (Narcissin)	[44]
17	4.26	287.0554 [M+H]^+^	−0.7	287[M+H]^+^ 241[M+H-H_2_O-CO]^+^ 213[M+H-H_2_O-2CO]^+^ 153 ^1,3^A^+^ 121 ^0,2^B^+^	Kaempferol	[31]
18	4.27	303.0517 [M+H]^+^	4	137 ^0,2^B^+^ 153 ^1,3^ A^+^ 229[M+H-H_2_O-2CO]^+^ 257[M+H-H_2_O-CO]^+^ 303[M+H]^+^	Quercetin	[31]
19	4.31	449.1081 [M+H]^+^	−0.7	449[M+H]^+^ 303[M+H-rhamnose]^+^ 285[M+H-rhamnose-H_2_O]^+^ 257[M+H-rhamnose-H_2_O-CO]^+^ 165 ^0,2^A^+^ 153 ^1,3^A^+^	Quercetin-3-O-rhamnoside (Quercitrin)	[31,36]
20	4.47	609.1804 [M+H]^+^	−2.5	286[M+H-Neohesperidose-CH_3_]^+^ 301[M+H-Rhamnose-glucose]^+^ 463[M+H-Rhamnose]^+^ 609[M+H]^+^	Diosmetin 7-O-neohesperidoside (Neodiosmin)	[45]
21	4.71	449.1077 [M+H]^+^	−1.6	449[M+H]^+^ 303[M+H-rhamnose]^+^ 257[M+H-rhamnose-H_2_O-CO]^+^ 153 ^1,3^A^+^	Quercetin-7-O-rhamnoside (Vincetoxicoside B)	[31,46]
22	5.01	493.1335 [M]^+^	−2.2	331[M-glucose]^+^ 493[M]^+^	Malvidin-3-O-glucoside	[47]
23	6.87	331.0809 [M+H]^+^	−2.7	273[M+H-2CH_3_-CO]^+^ 285[M+H-H_2_O-CO]^+^ 301[M+H-2CH_3_]^+^ 313[M+H-H_2_O]^+^ 316[M+H-CH_3_]^+^ 331 [M+H]^+^	Tricin	[31,48]
24	6.93	301.0717 [M+H]^+^	1.6	286[M+H-CH_3_]^+^ 301[M+H]^+^	Kaempferide(4’-O-methyl *kaempferol*)	[49]
25	7.89	269.0813 [M+H]^+^	−0.4	137 ^1,3^A^+^ 213[M+H-2CO]^+^ 237[M+H-CH_3_OH]^+^ 253[M+H-CH_3_-H]^+^ 254[M+H-CH_3_]^+^ 269[M+H]^+^	Formononetin Isoflavonoid	[47]
26	9.38	285.0761 [M+H]^+^	−0.7	285[M+H]^+^ 270[M+H-CH_3_]^+^ 242 [M+H-CH_3_-CO]^+^ 153 ^1,3^A^+^	Acacetin	[50]
27	9.39	285.0761 [M+H]^+^	−0.7	285[M+H]^+^ 270[M+H-CH_3_]^+^ 242 [M+H-CH_3_-CO]^+^	Calycosin	[51]
28	9.57	285.0757 [M+H]^+^	−2.1	133 ^1,3^ B^+^ 153 ^1,3^A^+^ 270[M+H-CH_3_]^+^ 285[M+H]^+^	Biochanin-A	[52]

## Data Availability

Not applicable.
